# Optimization Strategy of Traditional Block Form Based on Field Investigation—A Case Study of Xi’an Baxian’an, China

**DOI:** 10.3390/ijerph182010895

**Published:** 2021-10-17

**Authors:** Wei Feng, Wei Ding, Yingdi Yin, Qixian Lin, Meng Zheng, Miaomiao Fei

**Affiliations:** 1 School of Humanities and Social Science, Xi’an Jiaotong University, Xi’an 710049, China; 13991268970@126.com (W.F.); lingqixian1999@163.com (Q.L.); 2 School of Architecture and Urban Planning, Huazhong University of Science and Technology, Wuhan 430074, China; 3 School of Human Settlements and Civil Engineering, Xi’an Eurasia University, Xi’an 710065, China; yinyingdi@eurasia.edu; 4 Department of Architecture, School of Human Settlements and Civil Engineering, Xi’an Jiaotong University, Xi’an 710049, China; zhenmeng@xjtu.edu.cn; 5 China Qiyuan Engineering Corporation, Xi’an 710018, China; 18392021101@163.com

**Keywords:** microclimate, traditional block, block form, cold region

## Abstract

Rapid urbanization has caused environmental problems such as the urban heat island and air pollution, which are unfavorable to residents. Urban traditional blocks are facing the dual challenges of restoration and protection. This paper proposes adaptive transformation strategies for improving the microclimate of traditional areas. We selected Baxian’an Block in Xi’an city, simulated the air temperature and wind speed during summer and winter using ENVI-met, and studied the correlationship between morphological parameters (average building height, building density, enclosure degree, height fall, aspect ratio, and sky view factor) and air temperature and wind speed ratio. The case study revealed that the wind speed ratio of Baxian’an is relatively different in summer, reaching a maximum of 0.61, meaning that the ventilation capacity is significantly affected by the architectural form of the block. Finally, suggestions for the optimal design of the block’s form are provided: the building density should be less than 50%, the average building height should be more than 50 m, the enclosure degree should be less than 0.2, the height fall should be more than 41.7 m, and the sky view factor should be less than 0.5. This study can provide data and support for improving the planning and design standards of traditional residential areas.

## 1. Introduction

In developing countries, the protection and redevelopment of urban traditional blocks face many challenges [1,2]. The high density, height, and intensity of urban areas have led to a series of environmental problems that are harmful to human health, such as the frequent occurrence of the urban heat island (UHI) effect and the concentration of pollutants [3,4,5,6]. Traditional blocks are an important part of the city. In the process of the protection and redevelopment of traditional blocks, a comfortable and healthy urban outdoor environment and irreplaceable traditional heritage are of great importance to the whole city and residents [6,7,8,9].

Scholars have previously examined the influence of the wind environment on streets. Javanroodi et al. established three block cases that differed in terms of building density, shape, floor area ratio and height, etc. It was found that, at the micro scale, wind speed varied by 23% due to these factors [10]. In a comparison of seven urban forms, Wang et al. concluded that the wind speed with a lower building height area was larger than that with wide spacing between buildings [11]. Ng et al. discussed the correlation between the urban morphology and the wind environment using the morphological estimation method, and considered that the near surface permeability determines the wind speed [12]. Wang et al. used computational fluid mechanics (CFD) simulation to compare six typical urban cases, and found that there is a highly significant correlation between different urban forms’ layout and the wind environment [13]. Using the ideal city as a model and comparing two street layouts, Hang et al. concluded that the street layout would have different impacts on the surrounding wind environment [14]. Skote et al. found that when the wind blows over the city, the urban skyline and the overall morphology affects the wind flow [15]. Bouketta et al. found that the outdoor space has a significant relationship with the wind flow, and the aspect ratio has a large impact on the wind environment [16]. Tsichritzis et al. took London as an example and simulated it with CFD software. It was found that the urban facade area ratio was an important factor affecting the wind speed ratio [17]. Guo et al. found that closed urban blocks, rows of apartments, and a large number of buildings in the city are not conducive to the natural ventilation of the city [18]. Yuan et al. found that there is a certain correlation between tree morphology, urban density, and wind speed, so vegetation greening should be considered in urban planning and layout [19]. The above results provide considerations for wind environment optimization of traditional blocks in terms of the influencing factors of the block form.

Scholars have also discussed the influence of the thermal environment of the block. Xie et al. found that buildings in cities are mainly responsible for greenhouse gases [20]. Hart et al. found that the sky view factor (SVF), building footprint, and road lengths of equal divisions are the main factors affecting the air temperature [21]. Bumseok Chun’s research showed that green spaces can help cities cool in summer, and can also reduce the urban air temperature in winter [22]. Agnese Salvati found that the urban layout, vegetation coverage, human-generated heat inside buildings, and man-made traffic heat are important factors affecting urban air temperature [23]. Nassar et al. considered that the urban thermal environment in daytime is mainly affected by solar radiation, and the main influencing factors are BD, building height, and SVF of blocks [24]. Arifwidodo et al. demonstrated that the intensification of the UHI effect is caused by the increase in land use intensity [25]. Through the study of Xinjiekou Nanjing, Deng et al. found that good building layout can lead to cooling and effectively reduce the building energy consumption of blocks [26]. Chun et al. took Columbus, USA as a case study and found that solar radiation, urban space openness, greening rate, water area, and building roof area can affect the urban surface temperature [27]. Wang et al. found that different urban morphologies can result in different performances of the urban air temperature [28]. Based on cities in southern China, Tong et al. analyzed the influence of SVF, greening rate, sidewalk proportion, and building footprint on air temperature [29]. Taking Wuhan as an example, Huang and Wang discussed the effects of SVF, surface temperature, and different functional spaces on thermal environment from 2D and 3D perspectives in summer [30]. Similar to the wind environment research, the above research results also provide considerations for the optimization of the thermal environment of traditional blocks, particularly those relating to the differences of factors in different seasons.

However, the above research is not suitable for solving the problem of microclimate adaptability in traditional districts. Castaldo et al. discussed the air pollution and heat island problems of typical hilly traditional blocks in Italy [31] through field measurement and numerical simulation methods, and found that the main problem in traditional urban areas is the UHI effect. Abass discussed the measures used to mitigate heat islands in a traditional block of Bagdad, and found that the UHI effect can be reduced by using green and high-albedo building materials [32]. Ragheb et al. discussed the effects of air temperature, relative humidity, wind speed, and radiation temperature on human thermal comfort in a traditional block of Alexandria, Egypt, and provided suggestions for regional planning and design [33]. However, the optimization of the traditional plot form and the improvement of microclimate conditions have not been paid enough attention. Moreover, the comprehensive study of morphological parameters and a broad investigation of the reconfigurability of traditional districts have not been a focus of the existing studies.

This paper takes the Baxian’an traditional block in Xi’an, a typical basin city in China’s cold region, as the research object. The work can be summarized as follows: (1) analysis of the existing problems and the potential of block renovation through investigation and numerical simulation of the current situation; (2) study of the relationship between morphological parameters (average building height (ABH), building density (BD), enclosure degree (ED), height fall (HF), aspect ratio (AR), sky view factor (SVF), air temperature, and wind speed ratio; and (3) combination of the results of the parameter research and the investigation of the current situation of the block to propose block microclimate promotion strategies. This research can provide a reference for the redevelopment of traditional blocks in other countries and regions.

## 2. Methodology

### 2.1. Research Object

Xi’an is a typical low wind speed basin city with an annual air temperature of 13.7 °C, and an average wind speed of 1.9 m/s in summer and 1.4 m/s in winter. The dominant wind direction is east–northeast [34]. As the ancient capital of 13 dynasties in Chinese history, Xi’an city has a significant urban cultural heritage [35]. As a result of rapid urbanization, the preservation and innovation of the traditional style and features of this unique area is one of the most important problems for Xi’an city.

**Figure 1:**
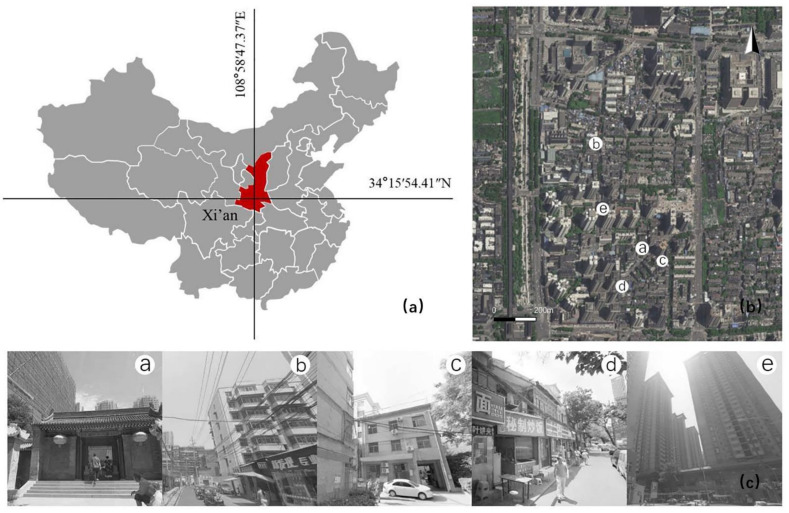
Location and status quo of the research site. (**a**) the location of Xi ‘an in China; (**b**) the location of research block relative to Xi’an city wall; (**c**) photos in different place according to the (**b**).

Located in the north of Xingqing Park and east of the east wall of Xi’an, Baxian’an Block was originally part of the site of Xingqing palace in the Tang Dynasty. Xi’an is the location of the largest Taoist temples. The development history of Baxian’an Block represents the development history of Xi’an City [36,37]. Unfortunately, the traditional block form is beginning to be gradually changed by modern construction activities. This study investigated and analyzed the status quo of architectural functional attributes, years of construction, and building height of the Baxian’an local block (location and current situation, as shown in Figure 1), explored the relationship between the architectural form and the thermal and wind environment of the block, and explored the renewal and transformation path of microclimate optimization of the traditional block.

### 2.2. Numerical Simulation

#### 2.2.1. Residential Type Distribution

**Figure 2:**
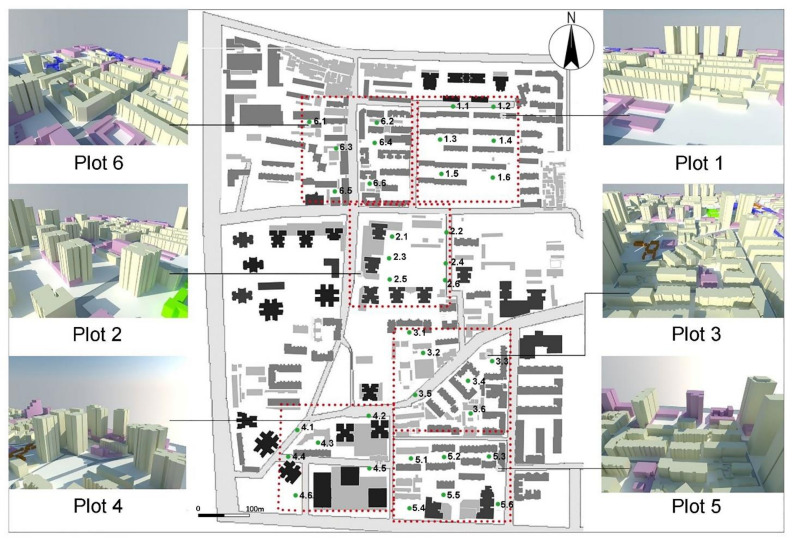
Residential type distribution of Baxian’an.

Before the simulation, the study site was classified according to the building form characteristics of the plot, and six typical residential areas were finally determined for analysis (Figure 2). Plot 1 corresponds to typical slab-type buildings, Plot 2 to enclosed high-rise buildings, Plot 3 to low-rise semi- enclosed buildings, Plot 4 to high-rise semi-enclosed detached buildings, Plot 5 to mixed high and low story buildings, and Plot 6 to low-rise enclosed buildings. Six monitoring points were placed in each plot (for example, point 1.1 of Plot 1 is written as P_1.1_), resulting in a total of 36 points, which functioned as air temperature and wind speed monitoring points. The meteorological parameters of the winter solstice and summer solstice in 2018 were used in the simulation.

#### 2.2.2. Simulation Software and Parameter Settings

ENVI-met was used to simulate wind and temperature fields. C.S. Gusson carried out an ENVI-met simulation and measurement in Sao Paulo (Brazil) and found that the simulated data of the software was very similar to the measured air temperature data [38].

According to the approaches of previous scholars, we considered the meteorological conditions of the urban airport data as the boundary input conditions of the software simulation [39,40,41]. As shown in Table 1, the simulation dates were 22 June 2018 and 22 December 2018 (the data used were from Xianyang International Airport of Xi’an, https://www.wunderground.com/history/daily/ZLXY/date/2018-6-22), and the 24 h simulation was carried out according to the hourly wind speed and air temperature of this weather station. The grid was set to x = 2 m, y = 2 m, z = 6 m, and the boundary value was set to 10. According to the survey, crowd activity is the most active between 8:00 and 18:00, so the results of this time period were selected for discussion.

The thermal environment and wind environment of six typical residential blocks in summer and winter solstice were simulated, and the air temperature and wind speed ratio of 6 monitoring points at the 1.5 m pedestrian level of each plot were analyzed during the period of 8:00–18:00. Combining the research scheme of Nanjing Xinjiekou and the investigation of Xi’an block morphology, we carried out the simulation study of idealized parameters as discussed in Section 2.3 [6,42].

### 2.3. Selection, Range Determination and Calculation of Parametric Research Factors

#### 2.3.1. Range of Variation and Calculation of Building Density (BD)

**Figure 3:**
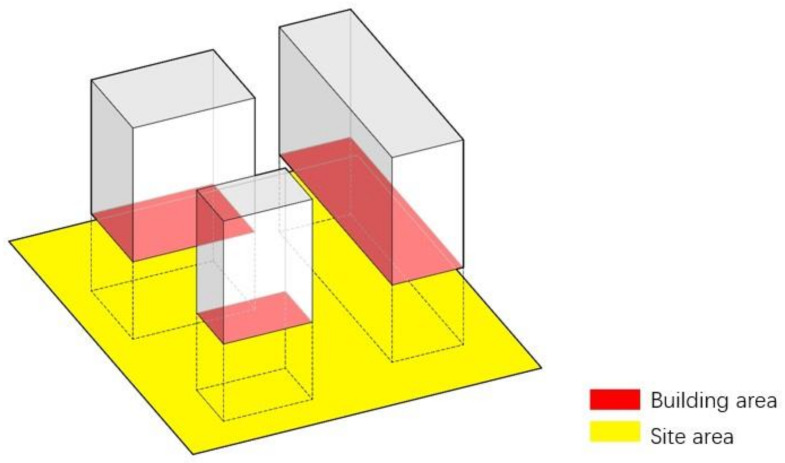
Calculation of BD.

BD refers to the proportion (%) of the total base area of buildings and the occupied land area within a certain range. It can reflect the open space ratio and BD within a certain land use range [43]. The calculation methods are shown in Equation (1) [44] and Figure 3. The correlation was judged by calculating air temperature and wind speed ratio with BD of 7.4%, 13.2%, 20.7%, and 29.8%.

Equation (1):

(1) $$\mathrm{Building}\;\mathrm{density}=\frac{\mathrm{The}\;\mathrm{total}\;\mathrm{base}\;\mathrm{area}\;\mathrm{of}\;\mathrm{buildings}}{\mathrm{The}\;\mathrm{occupied}\;\mathrm{land}\;\mathrm{area}}\times 100\%$$

#### 2.3.2. Range of Variation and Calculation of Average Building Height (ABH)

**Figure 4:**
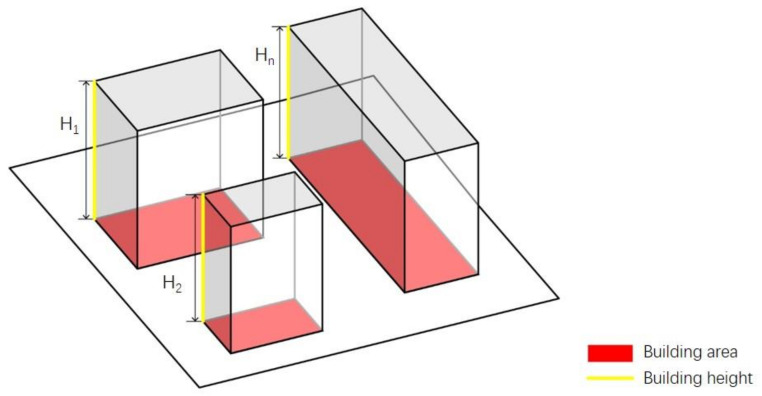
Calculation of average building height.

The ABH refers to the average height of all buildings in a certain area. Here, we use the weighted average of all buildings based on the building footprint to express the meaning of average height. The calculation method is shown in Equation (2) and Figure 4. The relationship between the ideal heights of 10, 20, 40, and 60 m, wind speed ratio, and air temperature is discussed.

Equation (2):

(2) $$\bar{H}=\frac{\sum_{i=1}^{n}S\cdot H}{\sum_{i=1}^{n}S}$$

$\bar{H}$ is the average height, *S* is the base area of the building, and *H* is the height of the building.

#### 2.3.3. Range of Variation and Calculation of Enclosure Degree (ED)

**Figure 5:**
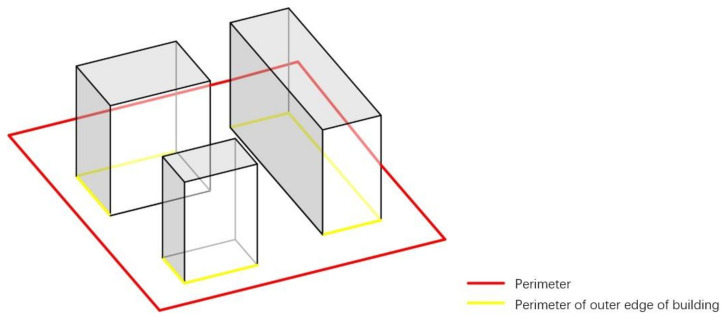
Calculation of ED.

ED refers to the ratio of the sum of the perimeter of all the outer buildings along the road to the perimeter of the outer edge of buildings within a certain range [45]. The calculation method is shown in Equation (3) [46] and Figure 5. When the ED is 0.20, 0.36, 0.57, and 0.82, the correlation between the ED and air temperature and wind speed ratio is discussed.

Equation (3):

(3) $$\mathrm{Enclosure}\;\mathrm{degree}=\frac{a_{1}+a_{2}+a_{3}+\ldots +a_{n}}{L}$$

where $a_{1},a_{2},a_{3},a_{n}$, represent the perimeter of the outer edge of the building in the calculation plot, and *L* represents the perimeter of the calculation plot.

#### 2.3.4. Range of Variation and Calculation of Height Fall (HF)

**Figure 6:**
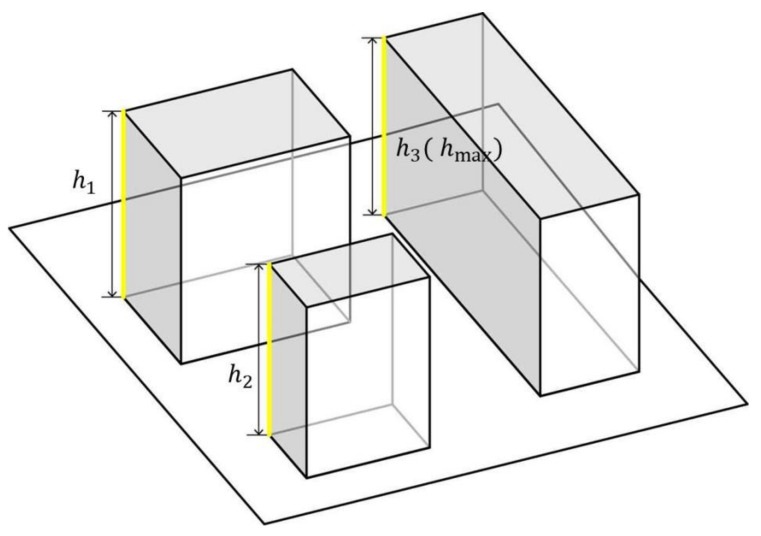
Calculation of HF.

HF represents the three-dimensional fluctuation characteristics of the urban spatial form, that is, the change in the HF in the vertical direction. The calculation method is shown in Equation (4) [6] and Figure 6. The mean wind speed ratio and air temperature variation of 0, 8.89, 23.3, and 41.7 were simulated and calculated.

Equation (4):

(4) $$\mathrm{Height}\;\mathrm{fall}\;=h_{max}-\bar{h}=h_{max}-\frac{h_{1}+h_{2}+\ldots +h_{n}}{n}$$

where $h_{max}$ represents the highest building height in the block, $\bar{h}$ represents the average building height in the block, $h_{1},h_{2},h_{n}$ represents the building height of each building, and *n* represents the number of buildings within the battery limit.

#### 2.3.5. Range of Variation and Calculation of Aspect Ratio (AR)

**Figure 7:**
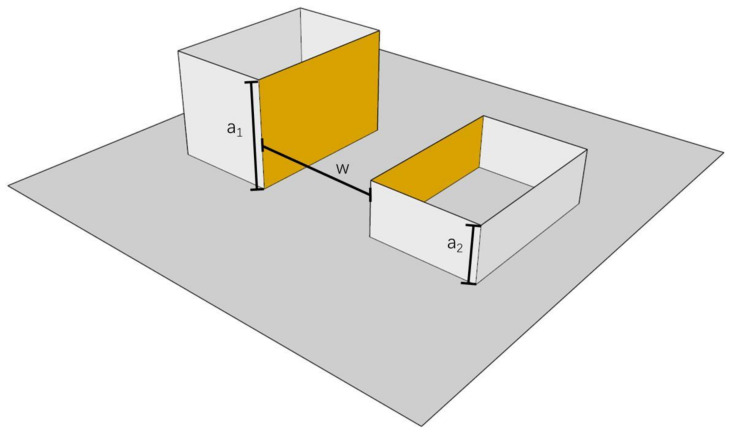
Calculation of AR.

AR is the average value of the sum of the buildings on both sides of the street divided by the width of the street. The calculation method is shown in Equation (5) [47] and Figure 7. In this study, the variation of air temperature and wind speed ratio was calculated when AR was 0.2, 0.3, 0.4, and 0.8.

Equation (5):

(5) $$\mathrm{AR}=\frac{a_{1}+a_{2}}{w}$$

#### 2.3.6. Range of Variation and Calculation of Sky View Factor (SVF)

The calculation method of SVF is shown in Equation (6). In this study, the wind speed ratio and air temperature variation were simulated when the SVF was 0.43, 0.32, 0.21, and 0.

Equation (6):

(6) $$\mathrm{SVF}=\frac{S_{Sky}}{S_{View}}$$

where SVF represents the sky view factor, $S_{Sky}$ represents the sky area within the field of view, and $S_{View}$ represents the full area of the 180° field of view.

## 3. Results

### 3.1. Current Situation of Baxian’an

#### 3.1.1. Different Building Functions

**Figure 8:**
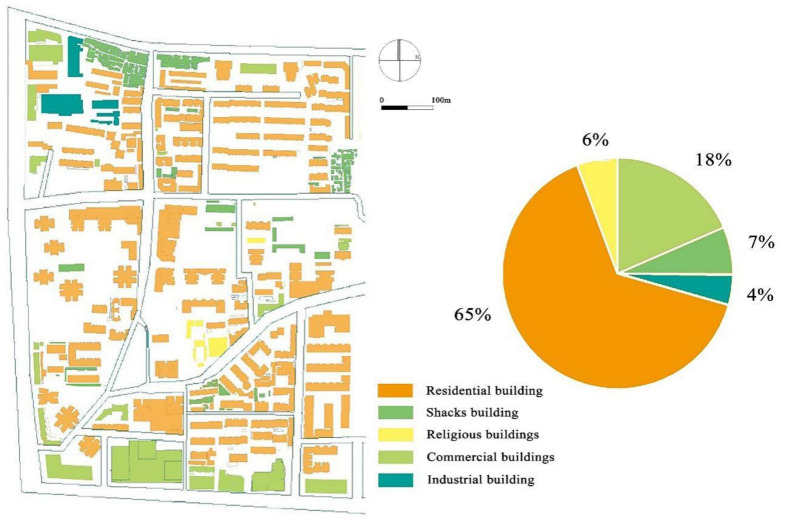
Architectural function distribution of Baxian’an.

The Baxian’an traditional and cultural district is rich in architectural functions, particularly residential and related functions. According to the investigation, the current situation of building function was drawn (as shown in Figure 8). In the study area, the southern side of the street comprises commercial buildings, and shacks are mainly located to the north and northeast. The temple buildings of the Wangji Temple are located in the middle of the block, and the surrounding residential buildings are low-rise residential buildings, which were developed earlier. There is an industrial building in the northwest of the block, and the remainder are all kinds of residential buildings. According to the field survey, the residential buildings in the study area account for 75% of the total, followed by commercial buildings, which account for 14%. Most of these are distributed along the street, and residential buildings are distributed in groups.

#### 3.1.2. Different Periods of Construction

**Figure 9:**
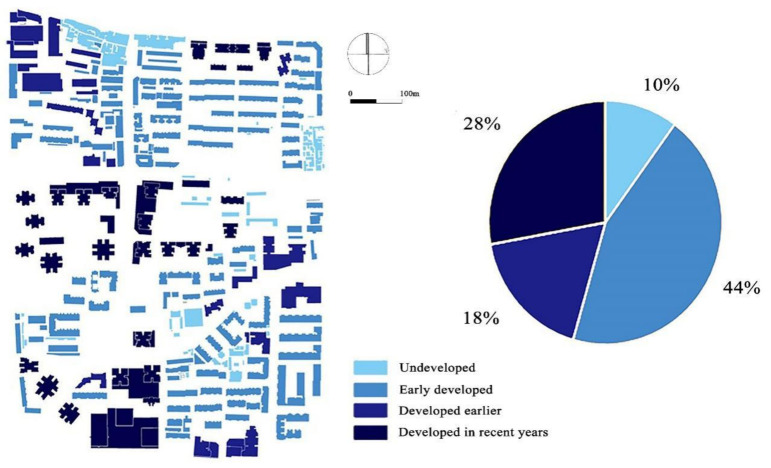
Distribution of architectural development periods of Baxian’an.

The buildings in Baxian’an Block were built in different times, and the level of development is complex. As shown in Figure 9, the proportion of undeveloped buildings is about 10%, the proportion of old houses developed in the early stage is the largest, reaching 44%, and the proportion of buildings developed in recent years is 28%. In the study area, there are residential areas with different periods and development levels, especially in high-rise residential areas. Although the surrounding infrastructure facilities of the residential area are complete, the BD and floor area ratio are high, the greening facilities are less, the building spacing is small, and the building volume including podiums is relatively large, which is not conducive to the ventilation of the whole community. At the summer solstice, the air temperature is higher at the pedestrian level.

#### 3.1.3. Different Heights of Construction

**Figure 10:**
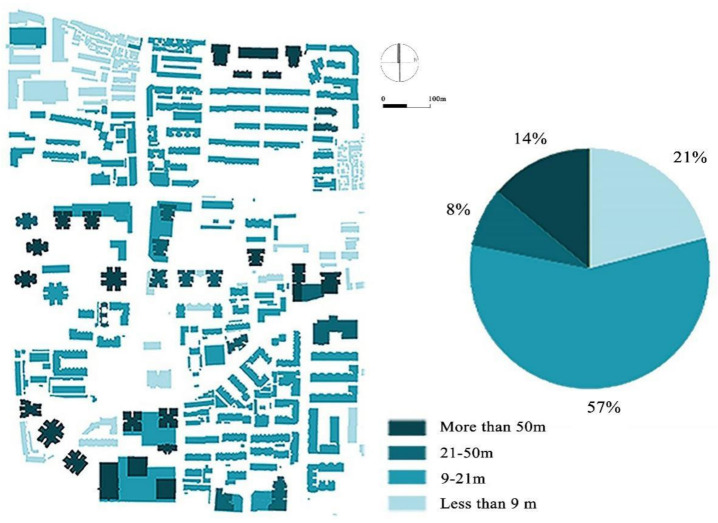
Building height distribution of Baxian’an (Feng W, 2020).

As shown in Figure 10, the building heights in Baxian’an Block are different. About 21% of the buildings in the study area have a height of 1–9 m and were mostly built by shack residents. The buildings with a height of 10–20 m account for 57%. These buildings are mostly residential buildings constructed under early plans. Buildings with a height of 20–50 m, which are between the low-rise and the high-rise buildings, account for only 8% of the total number. Most of these were built between 2000 and 2010. The buildings above 50 m account for 5%, and were mostly developed after 2000. These are mainly distributed in the south and middle of the block, whereas high-rise blocks are mainly distributed in the middle of the block. It is obvious that this results in a poor downwind ventilation effect.

#### 3.1.4. Potential Analysis of Block Renovation

The existing buildings in Baxian’an Block can be divided into self-built houses, traditional buildings, early buildings developed in the 1980s, and buildings developed in the past 10 years. The characteristics of these four types of architecture are as follows: (1) Undeveloped residents’ self-built houses with 1–6 floors, without considering lighting and ventilation, are chaotic and complex. These buildings have high reconstruction potential. (2) Traditional buildings, such as temples, are necessary to maintain the original style, and must be partially repaired. (3) The “Tongzi” buildings were developed at a very early stage and were mostly inspired by the Russian architectural style in the 1980s, with little consideration of the external environment of the buildings. There is a high reconstruction potential for this kind of building, which occupies a large area in the whole block, and the improvement effect on the environment would be significant. 4) The buildings developed in recent years are mostly medium- and high-rise residential buildings. Their development level is relatively high, but their impact on the microclimate environment was not considered. These buildings account for a small proportion in Baxian’an Block, and have a large number of stories and a high reconstruction cost.

### 3.2. Numerical Simulation

#### 3.2.1. Correlationship between Low-Rise Building Forms and Wind Speeds and Air Temperature

(1) Slab-type buildings

**Figure 11:**
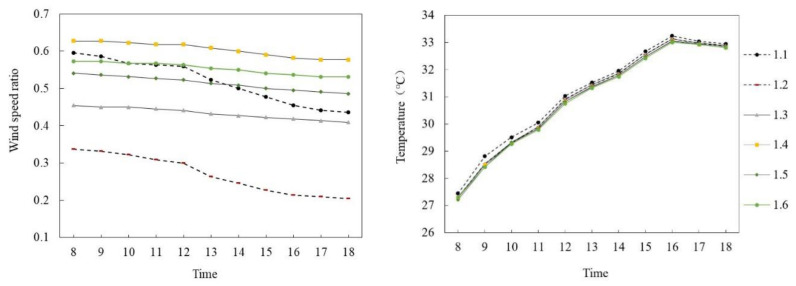
Wind speed ratio and air temperature simulation of slab-type buildings on the summer solstice.

**Figure 12:**
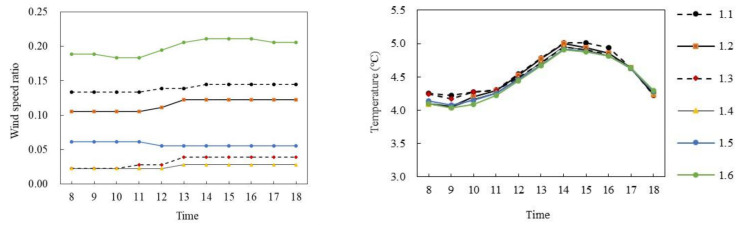
Wind speed ratio and air temperature simulation diagram of slab-type buildings (on the winter solstice).

Figure 11 and Figure 12 show the simulated results of the air temperature and the wind speed ratio of slab-type buildings in summer and winter, respectively. The typical days from 8:00 to 18:00 of six selected monitoring points in different space types were analyzed.

Figure 11a,b shows the wind speed ratio and air temperature simulation data of slab-type buildings on the summer solstice. In general, the wind speed ratio and air temperature trends of the six monitoring points at the site were the same during the period of 8:00–18:00, and the wind speed ratio difference was large but the air temperature difference was small. Specifically, the wind speed ratio of each monitoring point gradually decreased with time. The mean wind speed ratio of P_1.4_ was the largest, at 0.6, whereas that of P_1.2_ was the smallest, at 0.27, and the average difference was 0.33. The air temperature of each monitoring point first increased and then decreased with time. During 8:00–16:00, the air temperature of each monitoring point increased at about 0.71 °C per hour; P_1.1_ reached the maximum of 33.24 °C at 16:00 and then decreased at 0.15 °C per hour. The average air temperature difference between P_1.1_ and P_1.6_ was the largest, at 0.23 °C.

Figure 12a,b shows the wind speed ratio and air temperature simulation diagrams of the slab-type buildings on the winter solstice. Similarly, during the period of 8:00–18:00, with the exception of P_1.5_, the wind speed ratio of the other five monitoring points at the site had the same trend and the air temperature trend of each monitoring point was the same; however, the wind speed ratio difference was large and the air temperature difference was small. Specifically, the mean wind speed ratio of P_1.6_ was the largest, at 0.18, whereas that of P_1.4_ was the smallest, at 0.028, with an average difference of 0.152. In terms of air temperature, the air temperature of each point first increased and then decreased with time. During 8:00–14:00, the air temperature of each point increased by about 0.025 °C per hour; P_1.1_ reached the maximum of 5.01 °C at 14:00 and then decreased at 0.2 °C per hour. The average air temperature difference between P_1.1_ and P_1.6_ was the largest, at 0.1 °C.

(2) Low-rise semi-enclosed buildings

**Figure 13:**
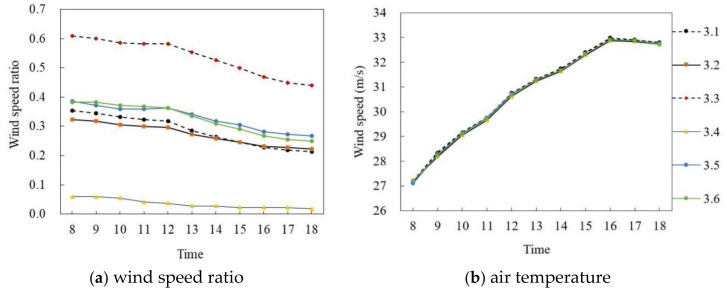
Air temperature and wind speed simulation diagrams of semi-enclosed buildings (on the summer solstice).

**Figure 14:**
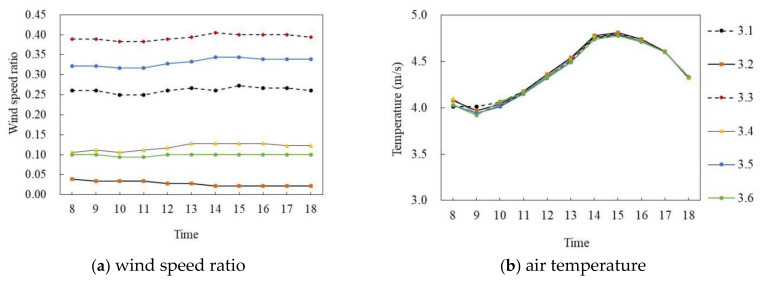
Air temperature and wind speed simulation diagrams of semi-enclosed buildings (on the winter solstice).

Figure 13 and Figure 14 show the simulation results of the air temperature and the wind speed ratio of low- and middle-rise enclosed buildings in summer and winter, respectively. Figure 13a,b shows the wind speed ratio and air temperature simulation diagrams on the summer solstice. In general, the wind speed ratio and air temperature trends of the six points at the site were the same during the period of 8:00–18:00; the wind speed ratio was quite different and the air temperature was almost the same. Specifically, the wind speed ratio of each point gradually decreased with time. The mean wind speed ratio of P_3.3_ was the largest, at 0.66, whereas that of P_3.4_ was the smallest, at 0.05, and the average difference was 0.61. The air temperature of each point first increased and then decreased with time. During 8:00–16:00, the air temperature of each point increased at about 0.72 °C per hour; P_3.1_ reached the maximum value of 32.97 °C at 16:00 and then decreased at 0.09 °C per hour. The average air temperature difference between P_3.4_ and P_3.1_ was the largest, at 0.1 °C.

Figure 14a,b shows the wind speed ratio and air temperature simulation diagrams on the winter solstice. During the period of 8:00–18:00, the wind speed ratios of the six monitoring points at the site had a large difference, and the air temperature difference was small. Specifically, in terms of wind speed ratio, the mean wind speed ratio of P_3.3_ was the largest, at 0.39, whereas that of P_3.2_ was the smallest, at 0.03, with an average difference of 0.36. The air temperature of each point first increased and then decreased with time. During 8:00–15:00, the air temperature of each point increased at about 0.093 °C per hour; P_3.2_ reached the maximum of 4.81 °C at 15:00 and then decreased at 0.16 °C per hour. The average value of each point was very close and the maximum difference in average air temperature was 0.03 °C.

(3) Low-rise enclosed buildings

**Figure 15:**
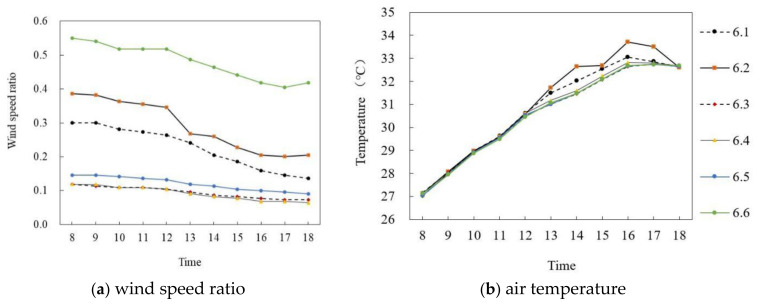
Air temperature and wind speed simulation map of semi-enclosed land in middle and low levels (on the summer solstice).

Figure 15a,b shows the wind speed ratio and air temperature simulation diagrams on the summer solstice. In general, the wind speed ratio of six monitoring points at the site had a large difference and the air temperature difference was small during the period of 8:00–18:00. Specifically, the wind speed ratio of each point gradually decreased with time. The mean wind speed ratio of P_6.6_ was the largest, at 0.477, and the mean wind speed ratio of P_6.4_ was the smallest, at 0.09, and the average difference was 0.387. In terms of air temperature, during 8:00–16:00, the air temperature value of each point increased at about 0.73 °C per hour; P_6.2_ reached the maximum of 33.7 °C at 16:00 and then decreased at 0.55 °C per hour. The difference in average air temperature between P_6.2_ and P_6.6_ was the largest, at 0.4 °C.

**Figure 16:**
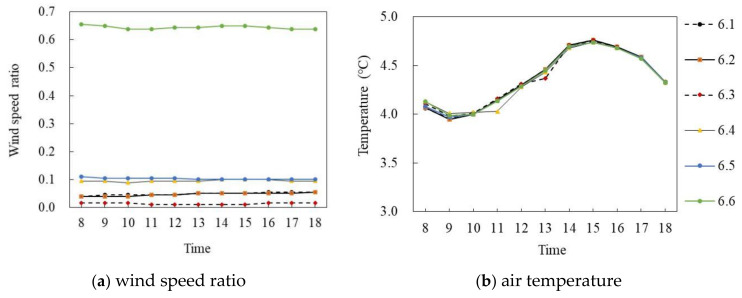
Air temperature and wind speed simulation map of the middle and low level semi-enclosed block (on the winter solstice).

Figure 16a,b shows the wind speed ratio and air temperature simulation diagrams on the winter solstice. During the period of 8:00–18:00, the wind speed ratio of six points at the site had a large difference, and the air temperature difference was small. Specifically, in terms of the wind speed ratio, the mean wind speed ratio of P_6.6_ was the largest, at 0.64, whereas that of P_6.3_ was the smallest, at 0.017, with an average difference of 0.627. In terms of air temperature, the air temperature value of each point first increased and then decreased with time. During 8:00–15:00, the air temperature of each point increased at about 0.09 °C per hour, and P_6.2_ reached the maximum value of 4.76 °C at 15:00, and then decreased at 0.14 °C per hour. The average value of each point was very close, at about 4.36 °C.

#### 3.2.2. Correlationship between Tall Building Form and Wind Speed and Air Temperature

(1) High-rise enclosed buildings

**Figure 17:**
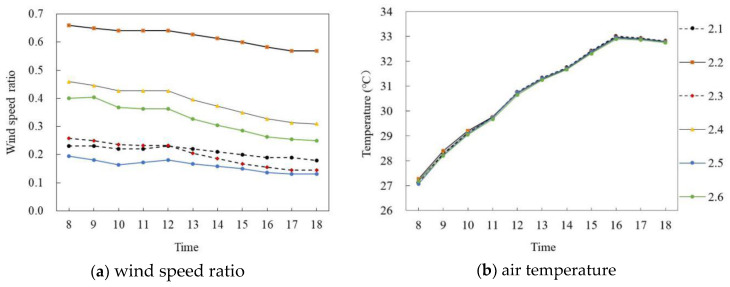
Air temperature and wind speed simulation diagrams of enclosed and high-rise detached buildings (on the summer solstice).

**Figure 18:**
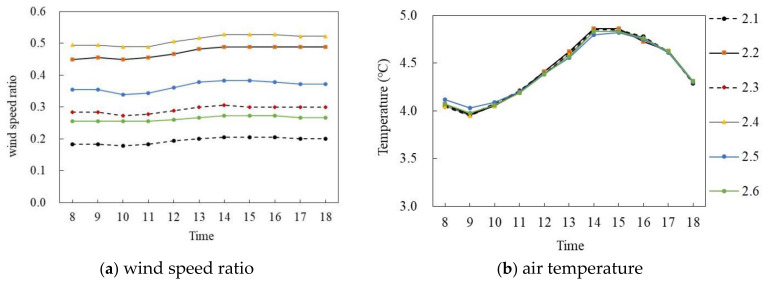
Air temperature and wind speed simulation diagrams of enclosed and high-rise detached buildings (on the winter solstice).

Figure 17 and Figure 18 show the simulation results of the air temperature and the wind speed ratio of high-rise enclosed buildings in summer and winter, respectively. Figure 17a,b shows the wind speed ratio and air temperature simulation on the summer solstice. In general, the wind speed ratio and air temperature trends of the six points at the site were the same during the period of 8:00–18:00; the wind speed ratio was quite different whereas the air temperature was almost the same. Specifically, the wind speed ratio of each point gradually decreased with time. The mean wind speed ratio of P_2.2_ was the largest, at 0.62, whereas that of P_2.1_ was the smallest, at 0.095, and the average difference was 0.525. In terms of air temperature, the air temperature of each point first increased and then decreased with time. During 8:00–16:00, the air temperature of each point increased at about 0.72 °C per hour; P_2.3_ reached the maximum of 32.98 °C at 16:00 and then decreased at 0.05 °C per hour. The average air temperature difference between P_2.2_ and P_2.6_ was the largest, at 0.13 °C.

Figure 18a,b shows the wind speed ratio and air temperature simulation on the winter solstice. Similarly, during the period of 8:00–18:00, the trends of the wind speed ratio and the air temperature of the six points at the site were the same; the difference in the wind speed ratio was large whereas the difference in the air temperature was small. Specifically, the wind speed ratio of each point gradually increased with time. The mean wind speed ratio of P_2.4_ was the largest, at 0.56, whereas that of P_2.1_ was the smallest, at 0.19, and the average difference was 0.37. The air temperature of each point first increased and then decreased with time. During 8:00–15:00, the air temperature value of each point increased at about 0.096 °C per hour; P_2.2_ reached the maximum of 4.86 °C at 15:00 and then decreased at 0.18 °C per hour. The average air temperature of each point was very close and the maximum difference in the average air temperature was 0.01 °C.

(2) High-rise and semi-enclosed buildings

**Figure 19:**
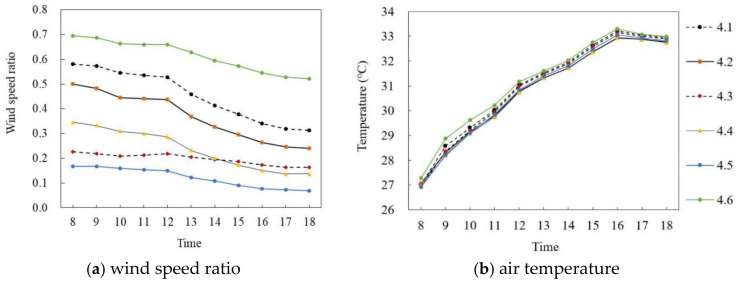
Air temperature and wind speed simulation of high-rise buildings (on the summer solstice).

**Figure 20:**
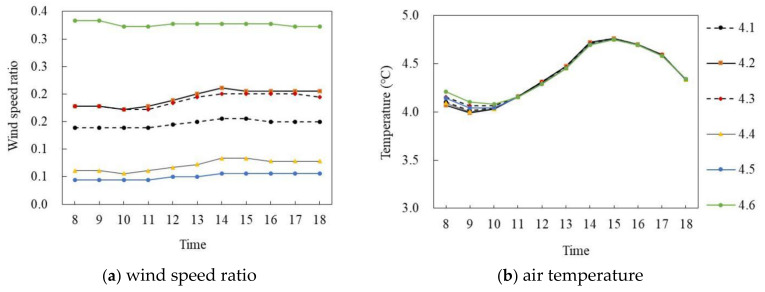
Air temperature and wind speed simulation of semi-enclosed buildings (on the winter solstice).

Figure 19 and Figure 20 show the simulation results of the air temperature and the wind speed ratio of high-rise semi-enclosed buildings in summer and winter, respectively. Figure 19a,b shows the wind speed ratio and air temperature simulation diagrams on the summer solstice. In general, the wind speed ratio and air temperature trends of the six points at the site were the same during the period of 8:00–18:00; the wind speed ratio was quite different and the air temperature was almost the same. Specifically, the wind speed ratio of each point gradually decreased with time. The average wind speed of P_4.6_ was the largest, at 0.61, and the mean wind speed ratio of P_4.5_ was the smallest, at 0.12, and the average difference was 0.49. In terms of air temperature, the air temperature of each point first increased and then decreased with time. During 8:00–16:00, the air temperature of each point increased at about 0.76 °C per hour; P_4.2_ reached the maximum value of 32.94 °C at 16:00 and then decreased at 0.12 °C per hour. The average air temperature difference between P_4.4_ and P_4.6_ was the largest, at 0.39 °C.

Figure 20a,b shows the wind speed ratio and air temperature simulation on the winter solstice. During the period of 8:00–18:00, the wind speed ratio of six points at the site had a large difference whereas the air temperature difference was small. Specifically, in terms of wind speed ratio, the mean wind speed ratio of P_4.6_ was the largest, at 0.34, and the mean wind speed ratio of P_4.5_ was the smallest, at 0.05, and the average difference was 0.29. In terms of air temperature, the air temperature of each point first increased and then decreased with time. During 8:00–15:00, the air temperature value of each point increased at about 0.09 °C per hour; P_4.1_ reached the maximum of 4.76 °C at 15:00 and then decreased at 0.14 °C per hour. The average air temperature of each point was very close and the maximum difference of the average air temperature was 0.02 °C.

#### 3.2.3. Correlationship between the Mixed High and Low Story Building Form and Wind Speed and Air Temperature

**Figure 21:**
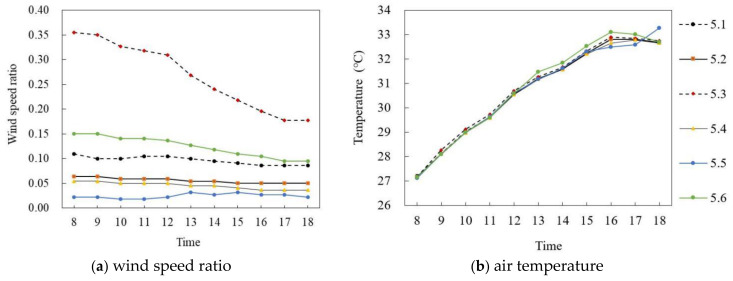
Air temperature and wind speed simulation of mixed high and low story buildings (on the summer solstice).

**Figure 22:**
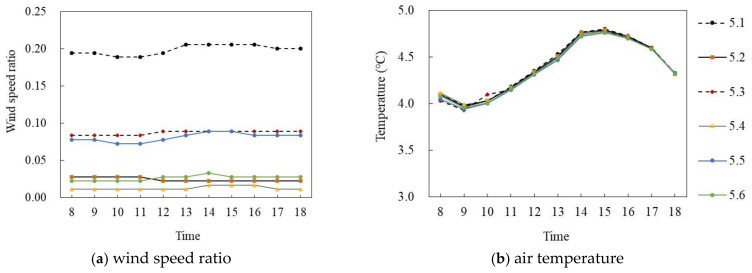
Air temperature and wind speed simulation of mixed high and low story buildings (on the winter solstice).

Figure 21 and Figure 22 show the simulation results of the air temperature and the wind speed ratio of mixed high and low story buildings in summer and winter, respectively. Figure 21a,b shows the wind speed ratio and air temperature simulation on the summer solstice. In general, the wind speed ratio of the six points at the site had a large difference and the air temperature difference was small during the period of 8:00–18:00. Specifically, the wind speed ratio of each monitoring point gradually decreased with time. The mean wind speed ratio of P_5.3_ was the largest, at 0.27, whereas that of P_5.5_ was the smallest, at 0.02, and the average difference was 0.25. In terms of air temperature, during 8:00–16:00, the air temperature of each point increased at about 0.7 °C per hour; P_5.6_ reached the maximum of 33.1 °C at 16:00 and then decreased at 0.21 °C per hour. The average air temperature difference between P_5.1_ and P_5.6_ was the largest, at 0.12 °C.

Figure 22a,b shows the wind speed ratio and air temperature simulations on the winter solstice. During the period of 8:00–18:00, the wind speed ratio of the six points at the site had a large difference and the air temperature difference was small. Specifically, in terms of wind speed ratio, the mean wind speed ratio of P_5.3_ was the largest, at 0.09, whereas that of P_5.4_ was the smallest, at 0.01, and the average difference was 0.08. In terms of air temperature, the air temperature of each point first increased and then decreased with time. During 8:00–15:00, the air temperature of each point increased at about 0.09 °C per hour; P_5.5_ reached the maximum of 4.77 °C at 15:00 and then decreased at 0.085 °C per hour. The average air temperature of each point was very close, and the maximum difference of the average air temperature was 0.02 °C.

### 3.3. Parameter Study

The above numerical simulation results show that, on both the summer and winter solstice, the wind speed ratio in the block was low and the wind speed ratio at different points was quite different. In addition, the air temperature in the afternoon was higher than that in the morning, especially in summer. Therefore, we carried out parameter studies to further explore the correlationship between general morphological parameters (ABH, BD, HF, and ED) and special morphological parameters (AR and SVF) with the air temperature and the wind speed ratio. The simulated season in the following discussion is summer.

#### 3.3.1. Effect of Different Building Densities

**Figure 23:**
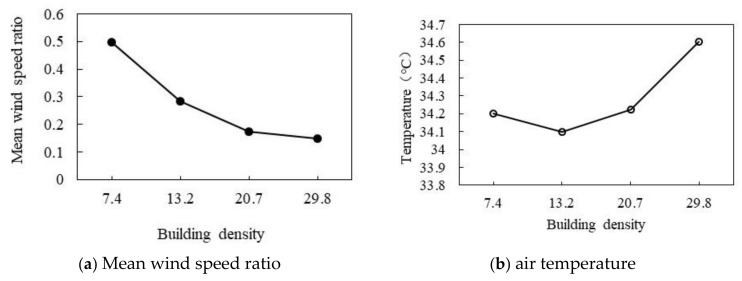
The correlation between air temperature, wind speed ratio, and BD.

As shown in Figure 23a,b, with the increase in BD, the wind speed ratio decreases, and the air temperature first decreases and then increases. As shown in Figure 23a, with the increase in BD, the wind speed ratio gradually decreases. When the average BD increases by 10%, the wind speed ratio decreases by 0.15. As shown in Figure 23b, with the increase in BD, the air temperature first decreases slightly and then increases gradually. When the BD is 13.2%, the air temperature decreases to the minimum of 34.1 °C. When the BD is 29.8%, the maximum air temperature is 34.6 °C.

#### 3.3.2. Effect of Different Average Building Heights

**Figure 24:**
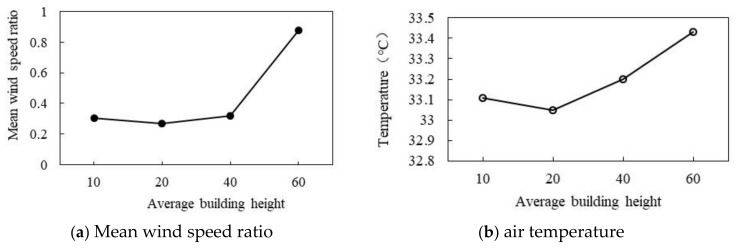
Correlationship between wind speed and ABH.

As shown in Figure 24a,b, the change in average height has the same effect on the air temperature and wind speed ratio. As shown in Figure 24a, wind speed ratio increases with the increase in average height. When the average height is 10 m, the wind speed ratio is 0.3. When the height is 20 m, the wind speed ratio reaches the minimum of 0.25, and then the wind speed increases with the increase in building height. When the average height is 60 m, the wind speed ratio reaches the maximum of 0.88. As shown in Figure 24b, the air temperature increases with the increase in average height. When the average height is 10 m, the air temperature is 33.11 °C. When the average height is 20 m, the air temperature reaches the minimum of 33.05 °C, and then increases with the increase in height. When the average height is 60 m, the air temperature reaches the maximum of 33.43 °C. In general, the wind speed ratio increases by 0.126 and the air temperature increases by 0.076 °C for every 10 m increase in ABH.

#### 3.3.3. Effect of Different Enclosure Degrees

**Figure 25:**
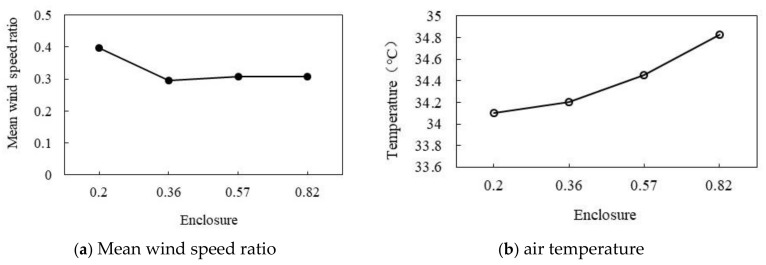
Correlation of wind speed and ED.

As shown in Figure 25a,b, the effects of the increase in ED on the wind speed ratio and air temperature are opposing. As shown in Figure 25a, with the increase in ED, the wind speed ratio gradually decreases. When ED is 0.2, wind speed ratio reaches the maximum of 0.4; when the ED degree increases to 0.36, the wind speed ratio decreases to the minimum of 0.295, and then the wind speed ratio is maintained at about 0.3. As shown in Figure 25b, the air temperature increases with the increase in the perimeter. When the perimeter is 0.2, the minimum air temperature is 34.1 °C, and then the average air temperature increases about 0.12 °C for every 0.1 increase in the ED. When the ED is 0.82, the maximum air temperature is 34.83 °C. The wind speed ratio decreases by 0.17 and the air temperature increases by 1.18 °C for each increase in the average ED.

#### 3.3.4. Effect of Different Height Falls

**Figure 26:**
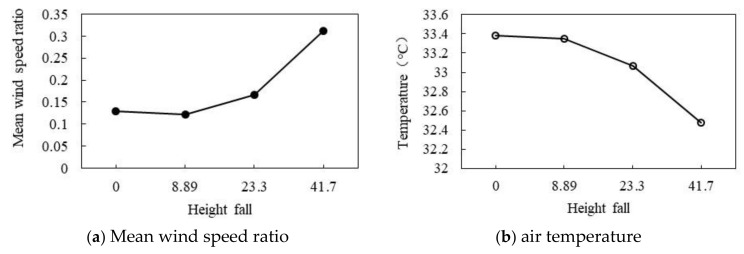
Correlation between wind speed and HF.

As shown in Figure 26a,b, when the HF is greater than 8.89, the effects of the increase in HF on the wind speed ratio and air temperature are opposing. As shown in Figure 26a, with the increase in HF, the wind speed ratio gradually increases. When the HF is 0, the wind speed ratio is 0.13. When the HF is 8.89, the minimum wind speed ratio is 0.12. When the HF is 23.3, the wind speed ratio is 0.17. When the HF is 41.7, the wind speed ratio reaches the maximum of 0.31. As shown in Figure 26b, the air temperature gradually decreases with the increase in HF. When the HF increases from 0 to 8.89, the air temperature decreases by 0.03 °C. When the HF increases from 8.89 to 23.3, the air temperature decreases by 0.29 °C. When the HF increases from 23.3 to 41.7, the air temperature decreases by 0.59 °C and reaches the minimum of 32.48 °C. In general, the wind speed ratio increases by 0.05 and the air temperature decreases by 0.22 °C for every 10 point increase in HF.

#### 3.3.5. Effect of Different Aspect Ratios

**Figure 27:**
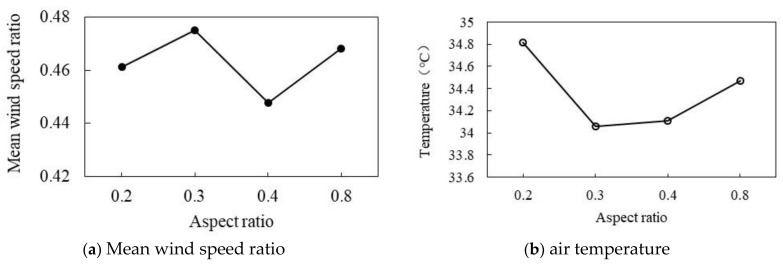
Correlation between wind speed and AR.

As shown in Figure 27a, the correlation between wind speed ratio and AR is small. When AR increases from 0.2 to 0.8, and wind speed ratio fluctuates around 0.46. As shown in Figure 27b, when AR is 0.2, the maximum air temperature reaches 34.82 °C, and when AR is 0.3, the air temperature reaches the lowest value of 34.06 °C. Subsequently, the air temperature increases with the increase in AR. In general, there is no simple linear correlation between AR and the wind speed ratio and air temperature.

#### 3.3.6. Effect of Different Sky View Factors

**Figure 28:**
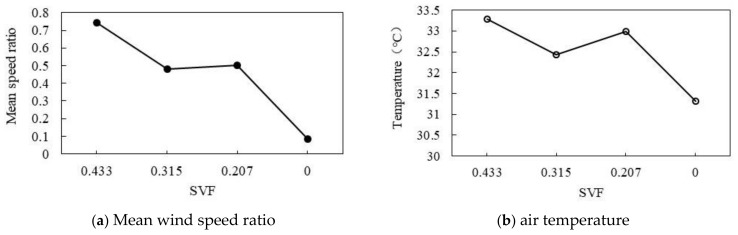
Correlation between wind speed ratio and air temperature and SVF.

As shown in Figure 28a,b, the decrease in SVF can simultaneously reduce the wind speed ratio and air temperature. As shown in Figure 28a, with the decrease in SVF, the wind speed ratio gradually decreases. When SVF is 0.433, the maximum wind speed ratio is 0.75. When SVF decreases from 0.315 to 0.207, the wind speed ratio is maintained at about 0.49. When SVF decreases to 0, the wind speed ratio reaches its lowest value of 0.09. As shown in Figure 28b, with the decrease in SVF, the air temperature decreases. When SVF is 0.433, the maximum air temperature is 33.29 °C. When SVF decreases from 0.32 to 0.207, the air temperature remains at 32.72 °C. When SVF is reduced to 0, the air temperature drops to the lowest value of 31.32 °C. Overall, the average SVF decreases by 0.1, the wind speed ratio decreases by 0.17, and the air temperature drops by 0.45 °C.

#### 3.3.7. Reanalysis of the Correlationship between Building Forms, Wind Speed Ratio, and Air Temperature in an Ideal State

Through the above analysis, the correlationship between the block building form parameters of the ideal building simulation results, and the wind speed ratio and air temperature, was obtained (see Table 2).

### 3.4. Morphological Parameter Design Strategy

According to the simulation results of the Baxian’an and parameters research, the wind speed ratio at different monitoring points is quite different, and the air temperature difference is not significant. Therefore, the building form design strategy is mainly aimed at improving the ventilation performance of the block. Based on the research of Yang et al. [6], and the Design Code for Heating and Air Conditioning of Civil Buildings in China [48], the good ventilation efficiency in this paper is confirmed as 0.6 (wind speed ratio ≥ 0.6). Accordingly, the BD should be less than 50%, the ABH should be more than 50 m, the ED should be less than 0.2, the HF should be more than 41.7 m, and the SVF should be less than 0.5.

## 4. Discussion

Some important results were obtained in this study, and further investigations are needed. The site survey showed that buildings with heights of 10–20 m account for 57% and residential buildings account for 75% of the total. The research area is located in Changle Fang, east of Xi’an City Wall, which is a typical area in which residents have lived and carried out religious activities for generations. Although the traditional characteristics of low-rise buildings are retained, part of the pattern is broken by modern high-rise buildings. Therefore, the results of this study may be more suitable for comparison with studies of building forms in residential areas. Numerical simulation showed that the wind speed ratio at different locations is significantly affected by the surrounding building environment and the wind direction. For example, in summer, the wind speed ratio of P_1.4_ was significantly higher than that of P_1.2_, which may be due to the fact that modern tall buildings block the inflow of the northwest wind, and the upwind direction of P_1.2_ is close to the buildings above 50 m. In the parameter study, the increase in BD led to a decrease in the wind speed ratio. In the direction facing the wind, as the area of obstruction increases, it becomes more difficult for wind to pass through the area. In addition, with the increase in ABH, the wind speed ratio decreased slowly and then increased significantly. This may be due to the wind being initially blocked and reduced, and the increase in building height can generate accelerated wind or turbulence, increasing the average wind speed. The buildings along the outer edge of the block are crucial to the flow field inside the block. There is no gap blocking the outer buildings, which reduces the air flow from the outside of the block to the inside of the block, and the ventilation effect between the buildings inside the block is significantly weakened. For example, in the parameter study, the increase in ED leads to the decrease in wind speed ratio. In this study, due to the setting of the simulated case, the wind environment had a more significant response to the change in the building shape than the thermal environment. Other scholars have also explored the relationship between block morphology and wind or thermal environments, as compared and discussed below.

In terms of wind environment, Shaeri et al. used CFD software to simulate the outdoor thermal environment of traditional blocks in Iran. The results showed that the wind speed ratios of open spaces and lane entrances are higher than those of other places [49]. The simulation results of Hu et al. indicated that when the residential BD increases from 0.18 to 0.32, the mean wind speed ratio of the residential area decreases by 0.18 [50]. Guo et al. considered that there was a negative correlation between ventilation efficiency and BD in Dalian City, and proposed measures such as using ventilation channels and increasing building height to improve urban ventilation performance [18]. In the study of Feng et al., it was found that SVF is positively correlated with the wind speed ratio, and the HF is positively correlated with the wind speed ratio [51]. Yang et al. also showed that when SVF increases by 10%, the wind speed ratio can increase by 7–8% [52]. Compared with other scholars’ studies, it was found that the relationship between morphological factors and the wind speed ratio is basically the same, but there are some differences in HF. Our conclusion is that, with the increase in HF, the block energy and the wind speed ratio increase because the change in the block height makes it easier for the wind to enter the block. The study came to the opposite conclusion probably because of the complexity of the real world.

In terms of thermal environment, Shareef et al. found that the diversity of building height can reduce the outdoor air temperature by 1.1 °C [53]. In the current study, the HF increased by 41.7, which reduced the average air temperature by 0.92 °C in the block. Yang et al. showed that there is no direct correlationship between the air temperature and BD [42], which is similar to the results presented in the current paper. In addition, in a study of Hong Kong City, Cheung et al. showed that the air temperature can be increased by 0.849 °C [54] by increasing SVF from 0 to 1. In the parameter study of Tong et al., it was shown that the maximum air temperature can be reduced by 0.05 °C [29] for every 10 m increase in building height. Compared with other studies, the temperature difference caused by the urban form presented in the current paper was smaller, which may be because we only considered the homogeneous surface of concrete blocks.

However, some limitations remain, as follows. Firstly, the scale of our research was at the block level. The influence of spatial planning factors on the urban climate at the urban scale will be the direction of our future research. In addition, the temperature and wind fields in boundary settings are in constant conditions under ideal conditions. In the future, further attention will be paid to the relationship between different climate zones and block form factors, in addition to the influence of different wind directions.

## 5. Conclusions

Based on the field investigation of Baxian’an Block, this study aimed to explore the promotion strategy of the microclimate in traditional residential areas. We carried out a numerical simulation of thermal and wind environments for the current situation of the block. Based on this, the idealized parameters were studied. The main conclusions are as follows:

(1) The layout of self-built and newly built buildings of the residents is chaotic, and the effect of the block form on the wind field is not fully considered in the block planning. Self-built buildings of residents and the buildings constructed in the 1980s have high potential for reconstruction.

(2) The architectural form design of the block has a significant influence on the ventilation efficiency. If the architectural form is not adjusted and optimized, the ventilation rate will not exceed 20%. The case simulation study of Baxian’an Block showed that the wind speed ratio at different monitoring points varies greatly (the maximum was 0.61).

(3) The case study showed that the mean wind speed ratio increases by 0.126 and the air temperature increases by 0.076 °C for every 10 m increase in ABH. The mean wind speed ratio increases by 0.05 and the air temperature decreases by 0.22 °C for every 10 point increase in HF. In addition, SVF decreases by 0.1, the mean wind speed ratio decreases by 0.17, and the air temperature decreases by 0.45 °C. It can be seen that the increase in building height, the space openness, and the building height drop in the block can enable more wind to enter the block interior. Building shading also has the potential to reduce the street temperature to some extent. However, ventilation and cooling need to reach a good balance in the buildings’ form control.

(4) Suggestions for the design of block form parameters are given as follows: the BD should be less than 50%, the ABH should be more than 50 m, the ED should be less than 0.2, the HF should be more than 41.7 m, and the SVF should be less than 0.5. However, in the process of transforming traditional blocks, the feasibility of microclimate adjustment should be considered comprehensively according to the actual demand and the influence mechanism of all parameters.

(5) This research can provide data support for the planning and design standards of the thermal and wind environments of residential areas in regions and countries around the world. It can also provide practical means to protect the common heritage of human civilization.

In addition, the following research areas need to be improved: (1) This study focused on the use of numerical simulation. In the future, field tests and questionnaires will be used to investigate the block microclimate. (2) This study took into account the effect of the difference in block morphology. The effect of the underlying surface and building surface materials will be considered in the future.

## Figures and Tables

**Table 1:** Parameter setting of ENVI-met simulation.

Summer		Winter	
Simulation time	0:00–24:00	Simulation time	0:00–24:00
Wind speed	2.2 m/s	Wind speed	1.8 m/s
Wind direction	315°	Wind direction	20°
Air temperature	23–39 °C	Air temperature	0–7 °C
Relative humidity	32–78%	Relative humidity	43–81%

**Table 2:** Summary of ideal state simulation results.

Factors	Wind Speed Ratio	Air Temperature
BD (building density)	The higher the BD, the smaller the wind speed ratio	The greater the density, the air temperature first decreases and then increases
ABH (average building height)	The higher the ABH, the wind speed ratio decreases first and then increases	The greater the height, the higher the air temperature
ED (enclosure degree)	The larger the ED, the smaller the wind speed ratio	The greater the ED, the higher the air temperature
HF (height fall)	The larger the HF, the greater the wind speed ratio	The higher the HF, the lower the air temperature
AR (aspect ratio)	The correlation is small	AR and the air temperature first decreases and then increases
SVF (sky view factor)	The larger the SVF, the greater the wind speed ratio	The larger the SVF, the lower the air temperature

## Data Availability

The data presented in this study are available on request from the corresponding author.
